# Effect of Quercetin Supplementation in Extender on Sperm Kinematics, Extracellular Enzymes Release, and Oxidative Stress of Egyptian Buffalo Bulls Frozen–Thawed Semen

**DOI:** 10.3389/fvets.2020.604460

**Published:** 2020-12-14

**Authors:** Ahmed R. M. El-Khawagah, Mohamed M. M. Kandiel, Haney Samir

**Affiliations:** ^1^Theriogenology Department, Faculty of Veterinary Medicine, Benha University, Toukh, Egypt; ^2^Theriogenology Department, Faculty of Veterinary Medicine, Cairo University, Giza, Egypt

**Keywords:** CASA, Egyptian buffalo, frozen semen, oxidative stress markers, quercetin, sperm kinetics

## Abstract

Buffalo spermatozoa are more sensitive for cryopreservation compared to other species. This study aimed to evaluate the consequences of quercetin against cryodamage of buffalo frozen–thawed spermatozoa characteristics. Semen of Egyptian bulls (*n* = 4) was extended in OptiXcell extender incorporated with quercetin at 0 (control), 2.5, 5.0, 10.0, 20.0, 40.0, and 80.0 μM before cryopreservation. Frozen–thawed semen was evaluated for sperm motility by computer-assisted sperm analyzer (CASA), viability, morphology, membrane, and acrosome integrities. The kinematics parameters including average path velocity (VAP; μm/s), straight linear velocity (VSL; μm/s), curvilinear velocity (VCL; μm/s), amplitude of lateral head displacement (ALH; μm), beat cross frequency (BCF; Hz), linearity [LIN, (VSL/VCL) × 100], and straightness [STR, (VSL/VAP) × 100] were assessed. The sperm-free extender was evaluated for aspartate aminotransferase (AST), alanine aminotransferase (ALT), and H_2_O_2_. Homogenized sperm cells were evaluated for oxidative stress biomarkers [superoxide dismutase (SOD), catalase (CAT), glutathione peroxidase (GPX)], and lipid peroxidation [malondialdehyde (MDA)]. The highest values of total motility, progressive motility, viability, intact acrosome, and membrane integrity substantially improved with 10 μM of quercetin. STR (%) was substantially low (*P* < 0.01), and VCL (μm/s) and ALH (μm) were markedly high (*P* < 0.05) in 10 μM of quercetin. The outflow of ALT enzyme to extracellular fluid was lower with 10 μM of quercetin (*P* < 0.001) and higher at 2.5 μM of quercetin. The spermatozoa leaked AST was markedly lower at 5.0, 10 (*P* < 0.001) and 20 μM (*P* < 0.05) of quercetin. The activity of antioxidant enzymes was eminently low at all quercetin concentrations, and this was accompanied by the decrease in H_2_O_2_ in the media. SOD activity at 10–80 μM, CAT at 5.0–40 μM, and GPX at 2.5–80.0 μM of quercetin in spermatozoa were substantially low. MDA level significantly (*P* < 0.001) decreased at all quercetin concentrations. In conclusion, the incorporation of quercetin at the level of 10 μM is promising in improving buffalo semen characteristics and lower the freezing–thawing oxidative stress.

## Introduction

Semen cryopreservation and its use in artificial insemination are beneficial policies for prolonged conservation of the hereditary material of superior bulls. Cryopreservation irreversibly damages spermatozoa resulting in their dysfunction. Despite the remarkable success in sperm cryopreservation technology, there is about a 50% decline in sperm motility after freezing and thawing (1). The loss in sperm functionality is attributed to the cryo-damages resulting from the harsh process of freezing and thawing (2). Several mechanisms have been claimed to be the main cause of sperm damage during the cryopreservation process including thermal shock (with intracellular and extracellular ice formation), cellular dehydration, and osmotic stress (3). It has been suggested that freezing and thawing of spermatozoa triggers the production of reactive oxygen species (ROS) (4). Although moderate levels of ROS are required for successful capacitation, acrosome reaction, and oocyte fusion (5), their overproduction during cryopreservation leads to sperm dysfunction (4).

Buffalo spermatozoa are sensitive to lipid peroxidation, as they contain higher levels of polyunsaturated fatty acid (PUFA) that entails a nearly equal distribution of saturated (47.8%) and unsaturated (49.8%) fatty acids (6). The lipid composition of the sperm plasma membrane has been stated as a major determinant of viability, motility characteristics, and membrane integrity (7, 8). Although seminal plasma provided some protection against peroxidation through its antioxidant's contents (9), semen dilution reduces the antioxidant availability for sperm. Therefore, the incorporation of antioxidants into semen extender is beneficial (10). Various antioxidants have been supplemented to different species (11–14) to counteract the adverse effects of ROS on sperm characteristics.

Quercetin (C_15_H_10_O_7_; molecular weight, 302.236 g/mol) is a flavonoid antioxidant commonly present in foods such as fruits and vegetables, able to scavenge reactive species and hydroxyl radicals (15) as well as provide beneficial health effects including anticarcinogenic (16), anti-inflammatory (17), and antimicrobial properties (18). It is composed of two benzene rings connected by an oxygen-containing pyrene ring (19). The flavonoids membrane affinity depends on the side chain length, hydroxylation degree, and molecular configuration (20). Quercetin with five OH groups has been found to have a strong membrane interaction (21). The presence and location of the hydroxyl (–OH) substitutions and the catechol-type B ring make quercetin an effective antioxidant, which possesses more intensive ROS scavenger activity than vitamin E or C (22).

Beneficial antioxidant properties of quercetin on frozen–thawed semen characteristics have been reported through its incorporation in semen extenders in different animal species including bulls (23), horses (3), boars (24), rams (25), and bucks (26). The need to be added to buffalo spermatozoa is more critical compared to other species due to the high levels of PUFA that substantially affect the equal distribution of saturated and unsaturated fatty acids (6) during the freezing–thawing processes. This in turn decreases the outcome of the whole process of cryopreservation.

Previous literature revealed the beneficial effects of quercetin on parameters of sperm quality (progressive motility and integrity of sperm plasma membrane, acrosome, and DNA) in various animal species (23–27). However, kinematics parameters of sperm, biochemical assessment of oxidative stress biomarkers in the spermatozoa homogenate, and the sperm free extender were not investigated in the buffalo semen. The present study aimed to evaluate the role of quercetin in preventing the cryodamage to spermatozoa of buffalo bulls in the OptiXcell extender and to verify its influence on post-thawing spermatozoa kinematics, semen characteristics, and oxidative stress biomarkers.

## Materials and Methods

### Semen Collection and Evaluation

Four proven fertile Egyptian native breed buffalo bulls with age of 3.5 ± 1.2 years maintained at El Abbasia Artificial Insemination Center, Cairo, Egypt, were used in the current study. Semen samples were collected with an artificial vagina maintained at 42–45°C for 7 weeks. Two ejaculates were collected from each bull, one after another, once a week. The time interval between the first and last ejaculate did not exceed in the worst conditions 30–45 min, as the bulls were well-trained and had high libido. Semen was kept for 10 min in the water bath at 37°C before being evaluated for its quality and suitability for extension and preservation. Sperm activity was assessed microscopically at 100× (mass motility) and 400× (individual motility). Sperm concentration was measured with the Neubauer hemocytometer. Good-quality semen samples (motility, livability, and normal sperm morphology ≥60–65% and sperm concentration ≥900 × 10^6^/ml) from the four bulls were pooled to minimize the individual variability and to attain adequate semen for triplicates. Pooled semen (seven replicates) samples were split into aliquots each of 4 ml for further processing.

### Semen Processing

Semen samples were extended using the OptiXcell extender (Ref. 024385, IMV® United States) to provide a concentration of nearly 50.0 ± 5.0 × 10^6^ sperms/ml. The extended semen was divided into seven experimental groups supplemented with different concentrations of quercetin to the extender (CAS Number 117-39-5, Sigma-Aldrich, United States) including 0 (control), 2.5, 5.0, 10.0, 20.0, 40.0, and 80.0 μM. These concentrations were selected after three preliminary trials in which we used different concentrations of quercetin (ranged from 50 to 200 μM) based on previously published works in bulls, rams, and bucks (2, 25, 28). All concentrations above 100.00 μM were found to be toxic to sperm cells and associated with complete cessation of sperm motility. The extended semen was slowly cooled (~2 h) to 4°C and was mechanically filled into 0.25 ml polyvinyl straws (Minitub, Germany) equilibrated for 2 h. After that, straws were racked horizontally in a vapor (5.5 cm above liquid nitrogen, N_2_) for 10 min before dipping and storage in the liquid N_2_ until assessing after 1 week. For evaluation, frozen straws were thawed (*n* = 5 per each trial per each concentration) in the water bath at 37°C for 40 s.

### Post-thawing Frozen Semen Assessment

#### Assessment of Sperm Motility and Kinematics Parameters

After thawing, the semen was incubated at 37°C, and the motility and kinematic parameters were evaluated using the computer-assisted sperm analyzer (CASA; Hamilton Thorne, Inc., Beverly, MA, United States) with a 10× objective at 37°C. Ten microliters of extended semen specimens were put onto a pre-warmed Makler chamber and evaluated. Motility values including total and progressive motility were recorded in percentages. The kinematics parameters including average path velocity (VAP; μm/s), straight linear velocity (VSL; μm/s), curvilinear velocity (VCL; μm/s), amplitude of lateral head displacement (ALH; μm), beat cross-frequency (BCF; Hz), linearity [LIN, (VSL/VCL) × 100], and straightness [STR, (VSL/VAP) × 100] were assessed. The sperm motilities were calculated with speed standards set as fast >80 μm/s, medium >60 μm/s, slow >20 μm/s, and static. For each evaluation, eight microscopic fields were randomly automatically selected and analyzed by the CASA system.

#### Assessment of Sperm Viability and Morphology

Slides for sperm viability and morphology were stained with eosin–nigrosin and examined microscopically at 1,000× magnification as previously described (29). Morphological abnormalities were determined as previously ascribed (30), where spermatozoa (*n* = 200/slide) were assessed for defects in the tail region including bent mid-piece, irregular mid-piece, broken tail, bent tail, coiled tail, and looped tail.

#### Assessment of Sperm Acrosome Integrity

Acrosomal membrane integrity was assessed using the Giemsa staining technique as described previously (31) with some modifications. Briefly, Giemsa stock solution was prepared by mixing 0.77 g Giemsa's powder (Merck) with a pre-warmed (40°C) 100 ml methanol and glycerol mixture (75 ml absolute methanol and 25 ml of glycerol) and saturated for 2–3 h after which the stain was filtered with 0.22 μm sterile Millex (Millipore). The solution was kept at 37°C in an incubator for 7 days in an amber color bottle with intermittent shaking.

After thawing, semen smears were prepared and air-dried on clean grease-free slides. The slides were fixed by dipping into a 5% formaldehyde solution at 37°C for 30 min. After fixation, the slides were removed out of the solution, washed under tap water, and air-dried for further processing. The working solution of Giemsa was prepared by mixing Giemsa's stock (3 ml), phosphate-buffered saline (PBS) (2 ml), and Milli-Q water (45 ml) in a cup and warmed at 37°C for 30 min. The smeared slides of spermatozoa were dipped into the working Giemsa solution and kept at 37°C for 2 h, after which the slides were removed and washed under tap water and air-dried. Spermatozoa (*n* = 200/slide) were examined microscopically at 1,000× magnification to assess the acrosomal intactness.

#### Assessment of Sperm Plasma Membrane Integrity

The hypoosmotic swelling test (HOS) was used to demark sperm plasma membrane integrity. The HOS solution (osmotic pressure ~190 mOsm/kg) was prepared from sodium citrate (Merck KGaA, Germany) 0.735 g and fructose (Merck KGaA, Germany) 1.351 g dispensed in 100 ml distilled water. To perform the assay, the semen sample (100 μl) was mixed with a pre-warmed HOS solution (900 μl) and incubated at 37°C for 60 min. Swollen and/or curled tails signified an intact plasma membrane, and accordingly, the percentage of HOS-positive sperms was calculated (32).

#### Assessment of Sperm Mitochondrial Activity

By using the 3′3 diaminobenzidine (DAB) assay, spermatozoa mitochondrial activity was assessed according to Hrudka (33). Briefly, semen was diluted at a ratio of 1:1 with 1 mg/ml solution of DAB in PBS and darkly incubated at 37°C for 1 h. After incubation, semen smears (10 μl) were prepared on microscope slides and air-dried. The slides were fixed for 10 min in 10% formaldehyde, washed, and air-dried again. Two hundred spermatozoa were counted using a phase-contrast optical microscope (1,000× magnification), and cells were classified into four categories: DAB I (100% of the midpiece was stained), DAB II (>50% of the midpiece was stained), DAB III (<50% of the midpiece was stained), and DAB IV (absence of staining in the midpiece).

### Semen Preparation for Biochemical Analysis

Frozen–thawed semen straws (*n* = 4 per each trial) from each group were centrifuged at 1,000× *g* for 20 min to separate spermatozoa from the semen extender. The supernatant containing the semen extender and seminal fluid was separated, labeled, and kept at −20°C until analysis (ALT, AST, H_2_O_2_). The spermatozoa were washed three times by suspending sperm pellets in an equal volume of cold fresh PBP and washed by centrifugation at 3,000× *g* for 10 min to remove the remaining extender and seminal plasma. The pellet was then suspended in 1.5 ml of ice-cold 5 mM Tris buffer, pH 8.0, and homogenized with a Polytron homogenizer for 15 s (34). The homogenate of the pellet was labeled and stored at −20°C until being assayed (CAT, GPx, SOD, MDA).

#### Biochemical Assessment of Spermatozoa-Free Extender for Enzymes Leakage

The activity of ALT (GPT113100, Egy-Chem for lab technology, Egypt) and AST (Ref. 260001, Spectrum, Egypt) were determined in a sperm-free semen extender colorimetrically at 365 and 546 nm, respectively, as it was described elsewhere (35). H_2_O_2_ was determined colorimetrically (at 510 nm) by the Phenol Red colorimetric method according to da Silva Maia et al. (36) using a commercial kit (HP 25, BioDiagnostic, Egypt).

#### Biochemical Assessment of Oxidative Stress Markers in Spermatozoa Homogenate

CAT (CA 2517, BioDiagnostic, Egypt), GPX (1.11.1.9, BioAssay Systems, United States), and SOD (SD 2521, BioDiagnostic, Egypt) were determined colorimetrically by a commercial kit as described formerly (37–39) at 520, 340, 560, and 534 nm, respectively. Lipid peroxidation (marked by MDA level) was evaluated in the sperm cell homogenate using commercial kits (MD 2529, BioDiagnostic, Egypt). Lipid peroxidation in spermatozoa was measured by the reaction of thiobarbituric acid (TBA) with MDA. The level of MDA was measured colorimetrically at 534 nm according to Ohkawa et al. (39).

### Statistical Analysis

Parameters were normally distributed using the Kolmogorov–Smirnov test. Data were tested for homogeneity of variances using Levene's test. Data of semen quality parameters (presented as mean ± SEM, *n* = 7), HOS (%), tail abnormalities (%), STR (%), LIN (%), VCL (μm/s), VAP (μm/s), ALH (μm), ALT (U/L), AST (U/L), H_2_O_2_ (mM/L), GPX (U/L), and MDA (nmol/ml) were analyzed with one-way analysis of variance (ANOVA) using SPSS (Ver. 23) and multiple comparisons of the means with Dunnett's test. Other parameters [total motility, progressive motility, viability (%), intact acrosome (%), VSL (μm/s), BCF (Hz), CAT (U/L), and SOD (U/ml)] showed significant non-homogeneity of variances, and therefore, they were analyzed using the non-parametric equivalent of the analysis (Kruskal–Wallis test and *post-hoc* Dunn test to compare between treatments). *P* < 0.05 indicates statistical significance.

## Results

### Effect of Quercetin on Sperm Survival Kinetics and Sperm Kinematics

**Figure 1:**
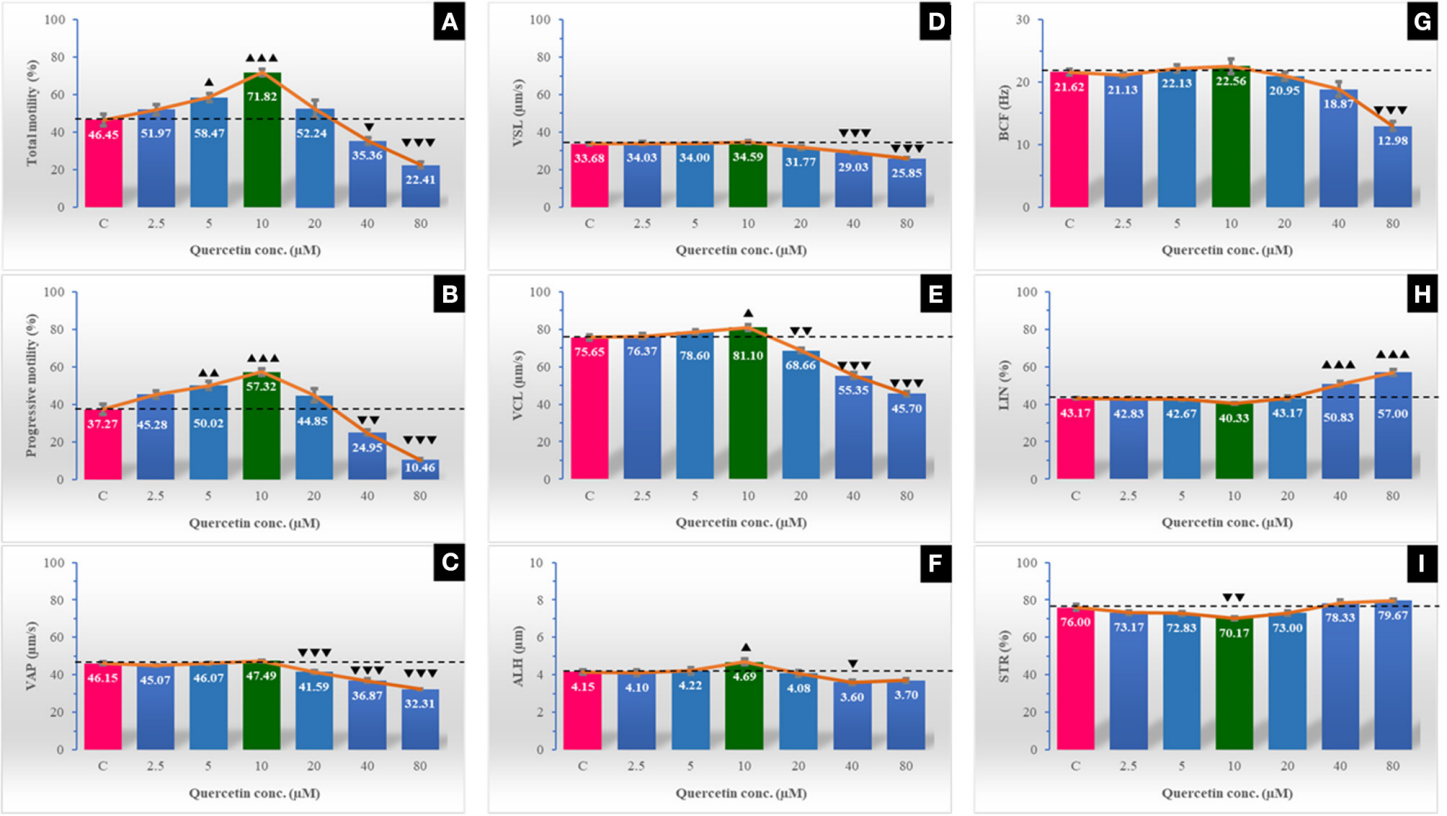
The effect of different quercetin concentrations on post-thawing sperm kinematics of Egyptian buffalo spermatozoa as measured by the computer-assisted sperm analyzer (CASA). The sperm kinematic parameters are total motility % **(A)**, progressive motility % **(B)**, average path velocity (VAP; μm/s) **(C)**, straight linear velocity (VSL; μm/s) **(D)**, curvilinear velocity (VCL; μm/s) **(E)**, amplitude of lateral head displacement, (ALH; μm) **(F)**, beat cross frequency (BCF; Hz) **(G)**, linearity (LIN) % **(H)**, and straightness (STR) % **(I)**. The horizontal dotted line delineates the average of the control group. The upper arrowhead (▴) indicated a positive impact. Low arrowhead (▾) indicated a negative impact. ▴, ▴▴, and ▴▴▴ referred to *P* < 0.05, *P* < 0.01, and *P* < 0.001, respectively. ▾, ▾▾, and ▾▾▾ referred to *P* < 0.05, *P* < 0.01, and *P* < 0.001, respectively.

**Figure 2:**
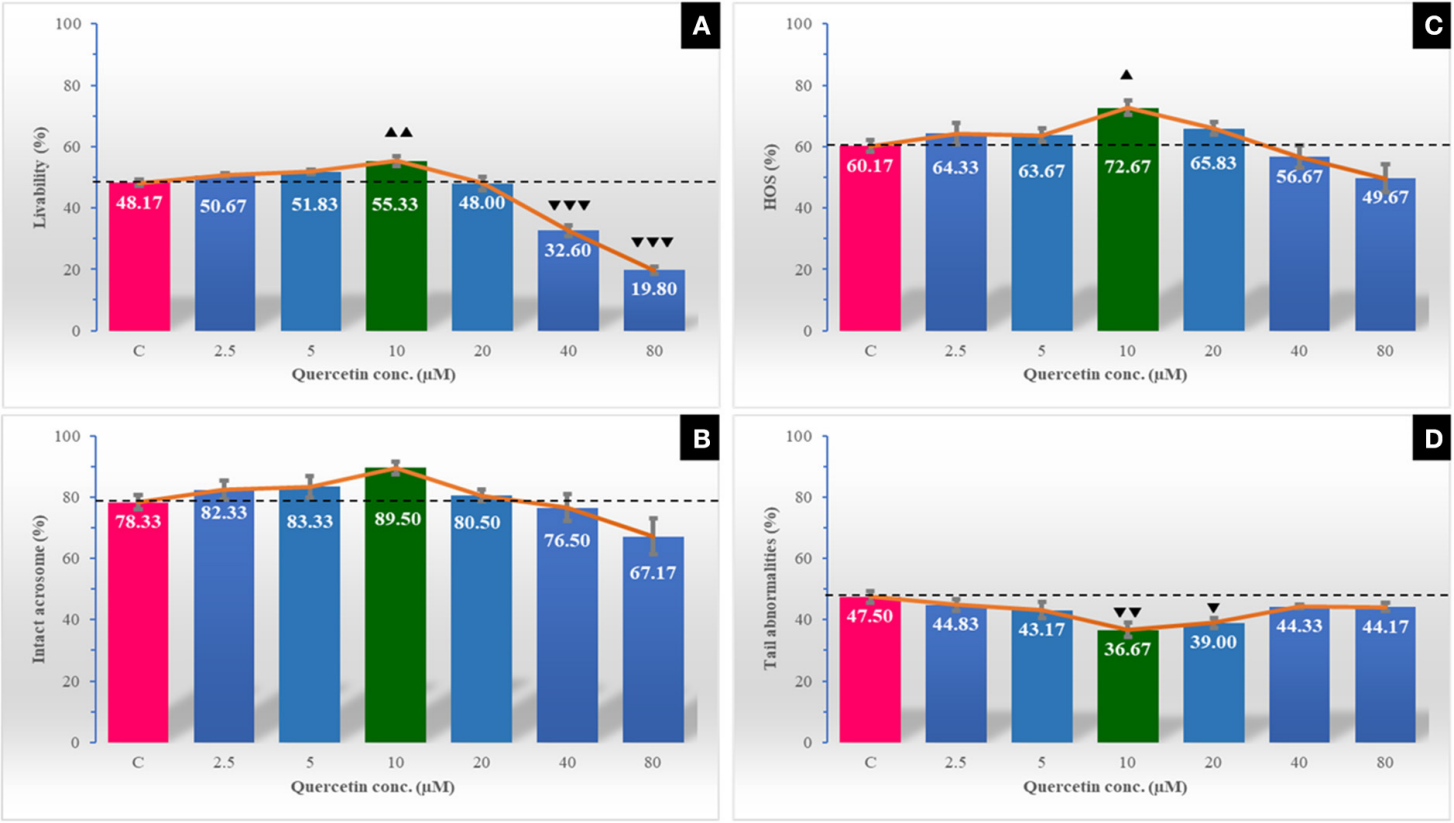
The effect of different quercetin concentrations on post-thawing semen characteristics [livability % **(A)**, intact acrosome % **(B)**, the percentage of positive sperms to the hypoosmotic swelling test (HOS %) **(C)**, and tail abnormalities % **(D)**] of Egyptian buffalo spermatozoa. The horizontal dotted line delineates the average of the control group. The upper arrowhead (▴) indicated a positive impact. Low arrowhead (▾) indicated a negative impact. ▴ and ▴▴ referred to *P* < 0.05 and *P* < 0.01, respectively. ▾, ▾▾, and ▾▾▾ referred to *P* < 0.05, *P* < 0.01, and *P* < 0.001, respectively.

**Figure 3:**
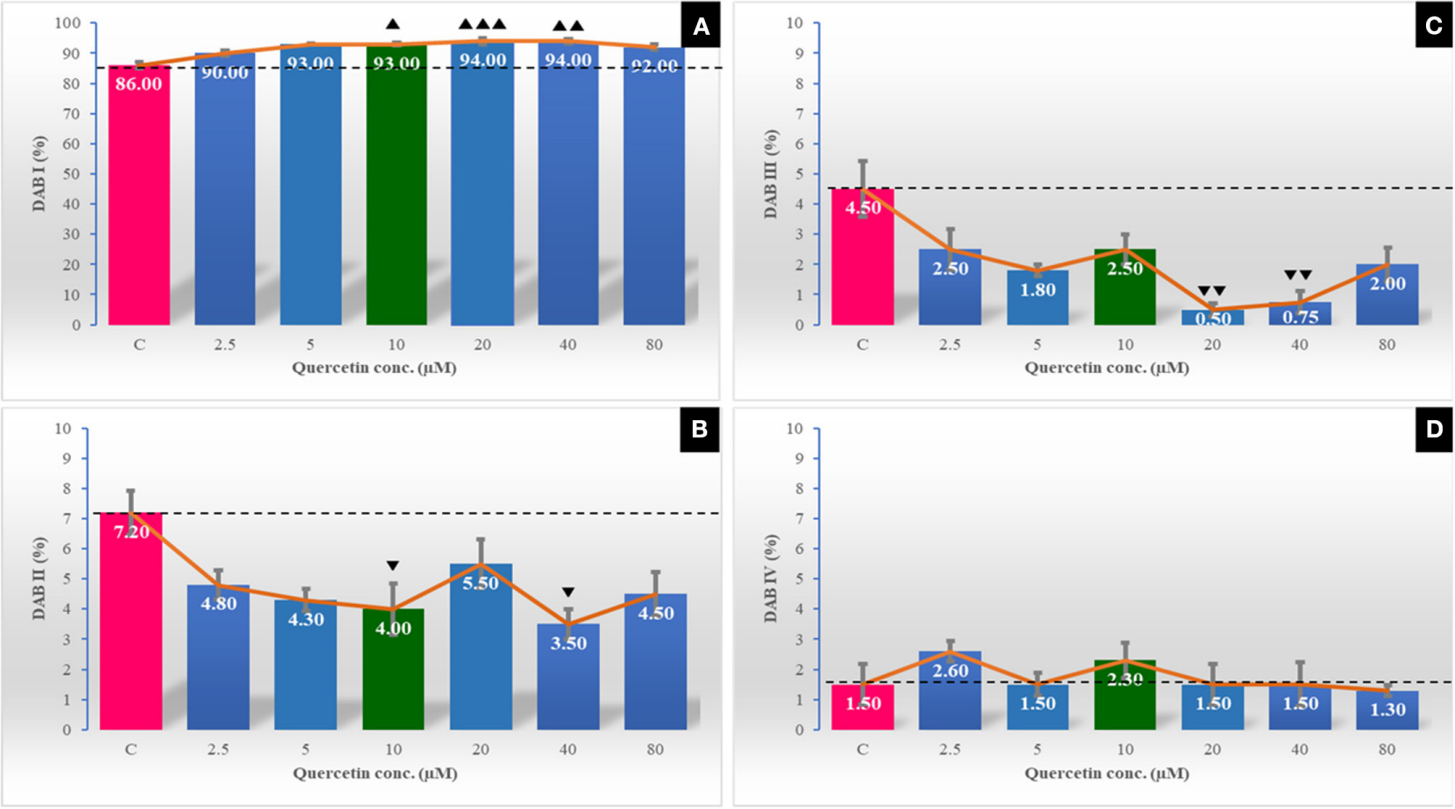
The effect of different quercetin concentrations on the mitochondrial activity as measured by the 3'3 diaminobenzidine (DAB) assay. Cells were classified into four categories: DAB I (100% of the midpiece was stained) **(A)**, DAB II (more than 50% of the midpiece was stained) **(B)**, DAB III (<50% of the midpiece was stained) **(C)**, and DAB IV (absence of staining in the midpiece) **(D)**. The horizontal dotted line delineates the average of the control group. The upper arrowhead (▴) indicated a positive impact. Low arrowhead (▾) indicated a negative impact. ▴, ▴▴, and ▴▴▴ referred to *P* < 0.05, *P* < 0.01, and *P* < 0.001, respectively. ▾, ▾▾ referred to *P* < 0.05 and *P* < 0.01, respectively.

The post-thawing characteristics of sperm survival kinetics and kinematics of buffalo were promising with the addition of quercetin at a rate of 2.5–20 μM (Figures 1, 2). The utmost values of total motility, progressive motility, viability, intact acrosome, and plasma membrane integrity substantially increased with the lowest percentage of tail abnormalities in the extender containing 10 μM of quercetin. STR (%) was substantially low (*P* < 0.01), while VCL (μm/s) and ALH (μm) were markedly high (*P* < 0.05) in 10 μM of quercetin. The highest concentrations of quercetin (40 and 80 μM) appeared to have a detrimental effect on sperm velocity parameters including VAP, VSL, and VCL, in addition to ALH (with 40 μM only) and BCF (with 80 μM only). Compared to control, mitochondrial activity showed a significant increase (*P* < 0.001) in DAB type I and a decrease (*P* < 0.01) in DAB types II and III, but non-significant changes in DAB type IV in quercetin supplemented groups were observed (Figure 3).

### Effect of Quercetin on Extracellular Transaminase Enzymes Release

**Figure 4:**
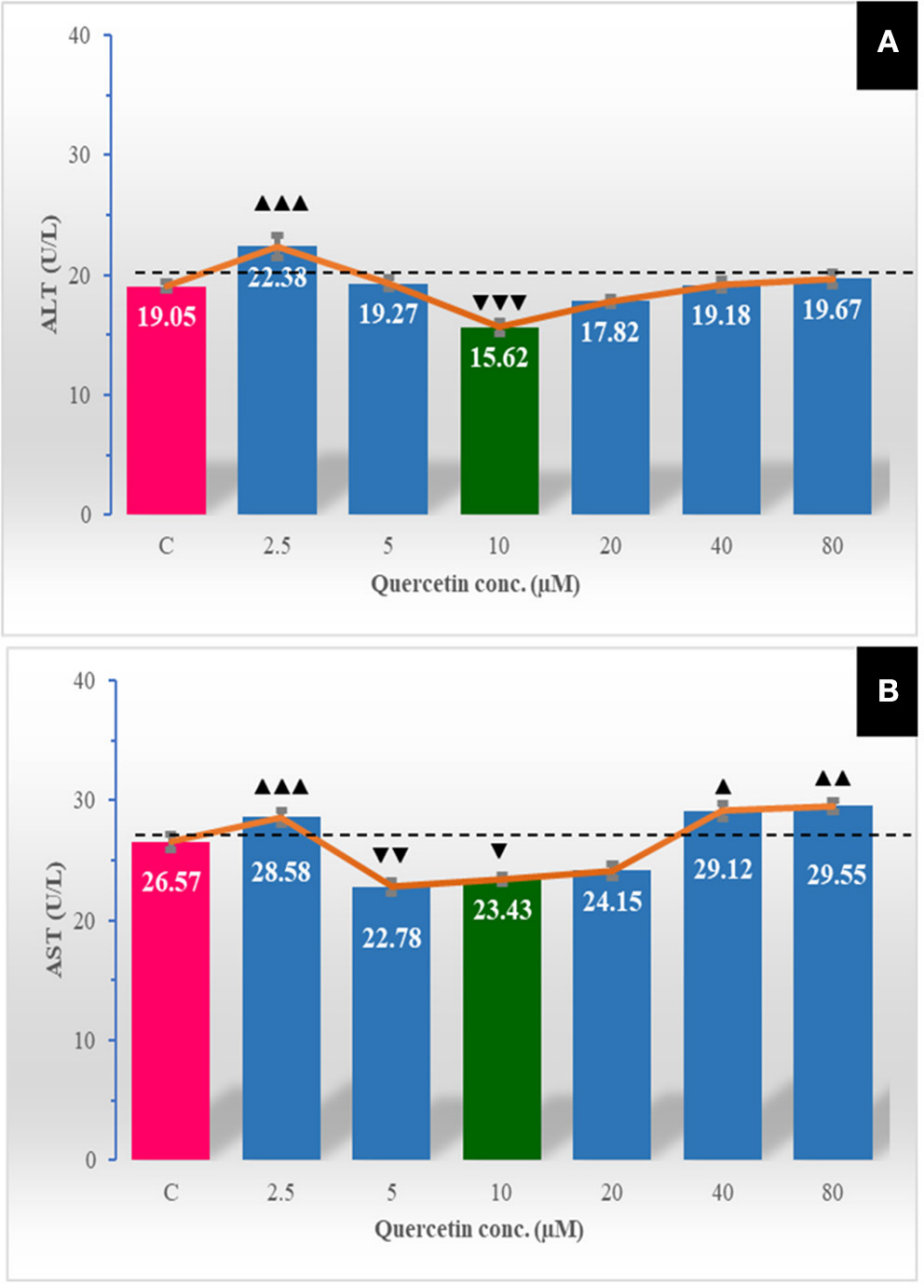
The effect of different quercetin concentrations on extracellular transaminases enzymes leakage [aspartate aminotransferase (AST; U/L) **(A)**, alanine aminotransferase (ALT; U/L) **(B)**]. The horizontal dotted line delineates the average of the control group. The horizontal dotted line delineates the average of the control group. The upper arrowhead (▴) indicated a positive impact. Low arrowhead (▾) indicated a negative impact. ▴, ▴▴, and ▴▴▴ referred to *P* < 0.05, *P* < 0.01, and *P* < 0.001, respectively. ▾, ▾▾, and ▾▾▾ referred to *P* < 0.05, *P* < 0.01, and *P* < 0.001, respectively.

A low level of quercetin (2.5 μM) was associated with an obvious (*P* < 0.001) increase in the escaped ALT enzyme to the extracellular media. Quercetin at a concentration of 10 μM markedly (*P* < 0.001) decreased the leakage of the ALT enzyme to sperm-free fluid (Figure 4). The leaked AST outside spermatozoa was markedly lower at 5.0 μM (*P* < 0.01) and 10 μM (*P* < 0.05) of quercetin but increased at 2.5 μM (*P* < 0.001), 40 μM (*P* < 0.05), and 80 μM (*P* < 0.01).

### Effect of Quercetin on Oxidative Stress Markers and Lipid Peroxidation

**Figure 5:**
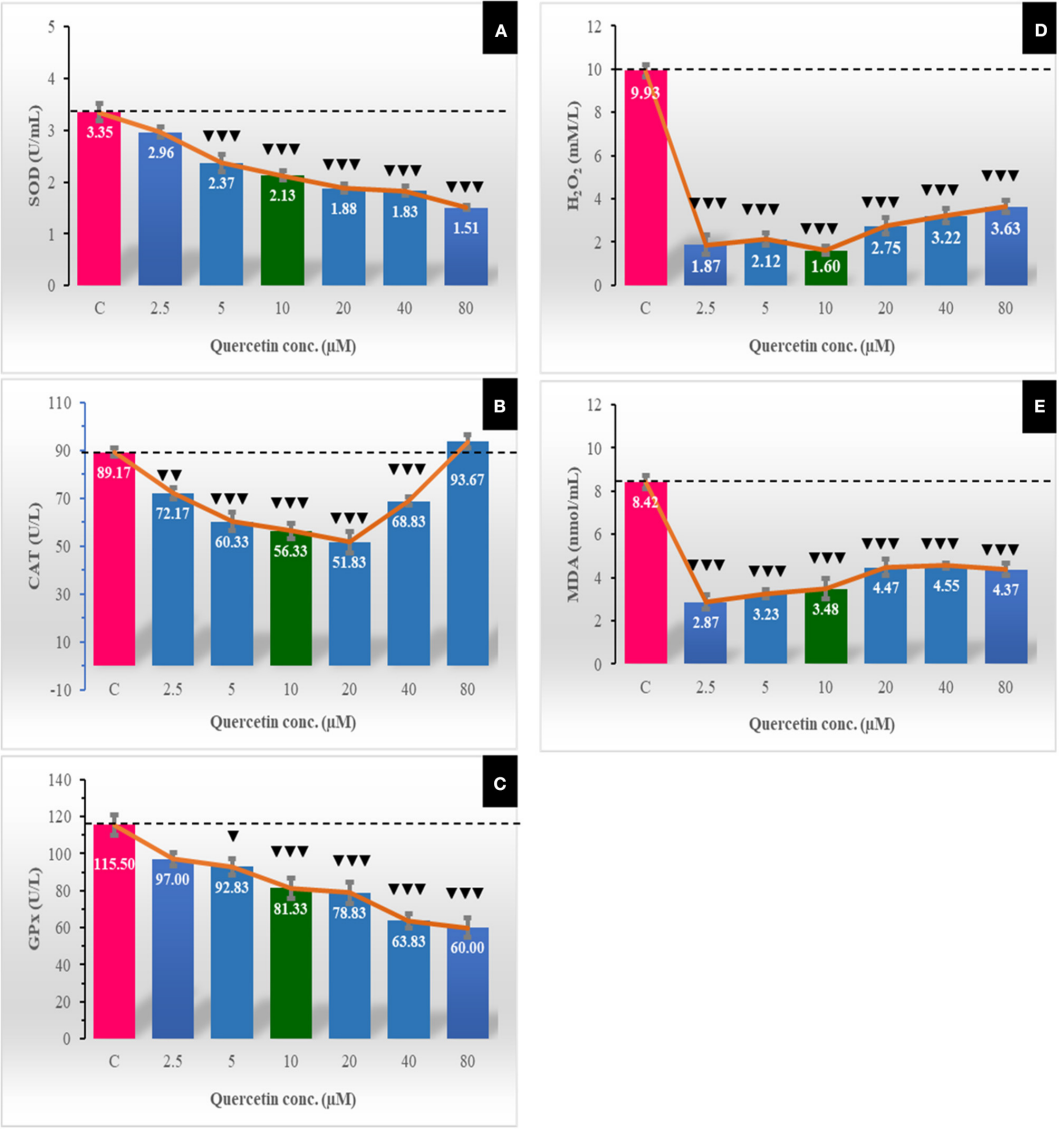
The effect of different quercetin concentrations on concentrations of some oxidative stress markers [superoxide dismutase (SOD; U/mL) **(A)**, catalase (CAT; U/L) **(B)**, glutathione peroxidase (GPX; U/L) **(C)**, H_2_O_2_ (mM/L) **(D)**, and lipid peroxidation (malondialdehyde (MDA; nmol/mL) **(E)**]. The horizontal dotted line delineates the average of the control group. The Low arrowhead (▾) indicated a negative impact. ▾, ▾▾, and ▾▾▾ referred to *P* < 0.05, *P* < 0.01, and *P* < 0.001, respectively.

The activity of antioxidant enzymes was eminently low at all quercetin concentrations, and this reflected on the decrease in hydrogen peroxide to the extender (Figure 5). SOD significantly (*P* < 0.001) decreased in a dose-dependent manner with increasing quercetin levels from 5 to 80 μM. CAT decreased at 5.0–40 μM of quercetin. GPX also showed the same dose-dependent decrease pattern as SOD. The levels of H_2_O_2_ and lipid peroxidation (MDA) were significantly (*P* < 0.001) lowered with all doses of quercetin compared to control.

## Discussion

Although it has great practicality, the cryopreservation process causes some damages to the sperm cells particularly because of ROS formation (4) and membrane lipid peroxidation (40), which have negative effects on sperm fertility parameters (41). In the present study, we tried to establish the optimal precooling concentration of quercetin in the extender used for cryopreservation of buffalo semen. To verify this, we assessed sperm cell survival and kinematic parameters as well as the antioxidative status of semen.

Motility of spermatozoa, especially the progressive motility, and velocity parameters are very important indices for the efficient fertilizing capacity of spermatozoa *in vivo* (42). The present study declared improving effects of quercetin (with concentration range of 2.5–20 μM) on sperm motility (total and progressive), with the highest values recorded at a concentration of 10 μM. Although sperm kinematics parameters (VSL, BCF, VCL, VAP, LIN, and VAP) were not changed at quercetin concentrations ≤20 μM, they were dramatically decreased at high doses of quercetin (40 and 80 μM). On the contrary, sperm VCL and ALH were significantly improved at 10 μM quercetin compared to the control. Our results agreed with that reported in goats (2). It was revealed in this species that quercetin with concentration of 10 μM improved sperm motility and velocity parameters. VCL (μm/s) measures the average velocity over the actual point-to-point track followed by the cell. This parameter is correlated positively with sperm fertilizing capacity (*R*^2^ = 0.67) (43), *in vitro* fertilization (44), and pregnancy (45) rates. ALH or amplitude of lateral head displacement is representing how fast a helical track segment of the spermatozoon revolves around its moving axis. The ALH is a measure of the lateral deviations of the spermatozoa head about its average path of progression and increases during hyperactivation (46). In addition, a positive effect of quercetin (at a concentration of 0.1 mM) on stallion sperm motility and velocity parameters was verified (3) compared to the high concentrations (0.2 and 0.3 mM). Moreover, a significant improvement was found in frozen–thawed ram sperm total motility with quercetin concentration at 10 μg/ml (47) and decreased with a higher concentration (50 μg/ml). In the same manner, Winn and Whitaker (42) recorded a significant improvement of frozen–thawed boar sperm progressive motility with the lower concentration of quercetin (0.25 mM) compared to higher concentrations (0.50 and 0.75 mM). The detrimental effects of the high concentration of quercetin that was reported in the present study were similar to those reported in bulls (28). On the other hand, Tvrda et al. (23) reported that quercetin concentrations ranging between 50 and 100 μM/L had a protective effect for bull sperm motility and mitochondrial activity against injury caused by lipid peroxidation of surplus ROS. The protective effect of quercetin on sperm motility may be related to its interaction with Ca^2+^-ATPase, a key enzyme involved in the regulation of sperm motility (48). Low concentrations of intracellular calcium are critical for supporting sperm motility, possibly by its role in intracellular cyclic adenosine monophosphate (cAMP) production (49). However, sustained elevation of calcium may suppress the mammalian sperm motility by lowering the cAMP concentration and restricting the ATP supply (50). Quercetin has inhibitory effects on the plasma membrane Ca^2+^-ATPase pump (51), resulting in an elevation of Ca^2+^ levels. Higher quercetin levels may induce higher inhibitory effects on the Ca^2+^-ATPase pump and higher Ca^2+^ levels, which adversely affect the sperm motility and velocity parameters (48).

Mitochondrial status plays an important role in sperm fertility through its relationship with the energetic status of sperm cells and motility (52). An inverse correlation has been reported between mitochondrial membrane potential (MMP) and ROS levels in frozen–thawed spermatozoa (53). Mitochondria are the major site of intracellular ROS formation. The coupling of electron transport with oxidative phosphorylation in sperm could be disrupted by ROS formation and reduced the number of sperm with normal mitochondria and sperm motility (54). Quercetin was demonstrated to be the most efficient compound in protecting against mitochondrial dysfunction by its ability to enter the cells and accumulation inside the mitochondria (55) and controlling the production of ROS by its antioxidant activity (28).

The integrity of the plasma membrane and viability of spermatozoa are very important predictors of its fertilizing capacity in buffalo (56). In the present study, quercetin (2.5–20 μM) had positive impacts on sperm viability and plasma membrane and acrosome integrities. The effect in the current study was dose dependent; the highest effect was being recorded with 10 μM, which produced the lowest level of sperm abnormalities. Our results agreed with that reported in the stallion semen, mainly with the lowest concentration of quercetin (3). Similar positive effects of quercetin were reported on the sperm viability in rams (47) and boars (24). On the opposite, high concentrations of quercetin (40 and 80 μM) were unfavorable to sperm viability and membranes integrities. These findings may be attributed to the prooxidant ability of the high doses of quercetin (3). Quercetin possesses the structural components of being an antioxidant, as it is characterized by a hydroxylation pattern of 3, 5, 7, 30, and 40 and a catechol B-ring (57, 58). However, quercetin may be converted into reactive products during exerting its antioxidant activity (59). It has been reported that quercetin oxidative degradation results in the formation of a free radical ortho-semiquinone intermediate, which may be converted to the parent compound or alternatively to an ortho-quinone, accompanied by ROS production such as superoxide and hydrogen peroxide (H_2_O_2_) (59). Therefore, the prooxidant activity of quercetin, particularly at high-dose levels, may be feasible and should be addressed (58).

The levels of the extracellular-leaked enzymes in the semen such as transaminase activities (AST and ALT) are good indicators of semen quality because they measure sperm membrane stability (60). Therefore, a high rate of sperm abnormalities causes sperm membrane damage and leakage of intracellular enzymes from spermatozoa and results in a high concentration of transaminase enzyme in the extracellular fluid (61). In the present study, quercetin at concentration 10 μM significantly decreased the level of leaked AST and ALT in the semen-free extender compared to the control, while the highest concentrations (40 and 80 μM) markedly increased the AST level compared to the other groups. These findings were concomitant with the results of membrane integrity and sperm abnormalities. The highest membrane integrity (at 10 μM of quercetin) preserved AST and ALT from leakage to the extracellular media; meanwhile, the lowest membrane integrity (at 40 and 80 μM of quercetin) leads to an increase in sperm-free media contents of ALT and AST.

Generally, the antioxidant system of the cell is composed of reduced SOD, CAT, glutathione, and glutathione peroxidase (62). Our results revealed that concentrations (5–40 μM) significantly decreased the level of catalase enzyme and H_2_O_2_. In the present study, the activity of SOD and concentration of GPX in spermatozoa decreased in all quercetin groups in a dose-dependent manner. Moreover, the lowest activity of catalase enzyme and H_2_O_2_ concentrations were recorded when the concentration of quercetin was 10 μM compared to other groups. The present results were similar to those reported in rabbit semen (63). Quercetin has been mentioned as a potent antioxidant because of its ability to inhibit ROS formation by enzymatic and non-enzymatic systems, especially reduced nicotinamide adenine dinucleotide phosphate (NADPH) oxidase (64) and nicotinamide adenine dinucleotide (NADH)-dependent oxidoreductase (65) that localized in the sperm plasma membrane and mitochondria (66). For lipid peroxidation, the present study revealed decreases in the levels of MDA in the quercetin groups compared to the control group. These results agree with that reported in humans (21), goats (2), rabbits (60), and stallions (67) spermatozoa. However, no effect of quercetin was found on the MDA level in frozen–thawed stallion semen (3). In the current study, lipid peroxidation was assessed by measuring the MDA in sperm cells (not in the extracellular fluid) to avoid the conflicting results that might be obtained due to lipid peroxidation of lipids in the extender. Thus, we assumed the existence of a direct relationship between the cellular lipid peroxidation and the intracellular content of quercetin, and we measured the MDA levels intracellularly.

## Conclusion

From the present study, we can conclude that quercetin is a potent antioxidant factor that could protect frozen–thawed buffalo spermatozoa from lipid peroxidation and oxidative stress. However, the concentration of quercetin supplementation in the extenders is a very important factor to be addressed. Supplementing the extenders with quercetin concentrations at 5–20 μM enhanced sperm motility, velocity parameters, sperm viability, and membrane integrities and prevented enzymes' outflow with marked improvement in the quality of the frozen–thawed spermatozoa at 10 μM concentrations. However, the highest concentrations of quercetin (≥40 μM) had harmful effects on most of the sperm parameters. Further investigations are necessary to confirm the fertilizing potential of sperm cells cryopreserved in quercetin supplemented extenders using a biological test such as functional *in vitro* fertilization (IVF).

## Data Availability Statement

The raw data supporting the conclusions of this article will be made available by the authors, without undue reservation.

## Ethics Statement

All the procedures were conducted according to the Ethics for the humane treatment of animal use in research guidelines and complied with the relevant legislation of the Faculty of Veterinary Medicine, Benha University, Egypt (BUFVTM 2/020720).

## Author Contributions

AE-K and MK designed the work and performed semen collection, processing, and post-thawing evaluation. HS helped in semen evaluation and writing the manuscript. All authors read and approved the final manuscript.

## Conflict of Interest

The authors declare that the research was conducted in the absence of any commercial or financial relationships that could be construed as a potential conflict of interest.
